# Comparison of the Periodontal Status of Patients Undergoing Labial and Lingual Orthodontic Therapy

**DOI:** 10.7759/cureus.6818

**Published:** 2020-01-30

**Authors:** V Vijaykumar, Vinoth Kumar R, Durvasulu Archana, Abinesh Sekar, Arun Deepak, Vivekanandhan Umapathy, Rajakumar P

**Affiliations:** 1 Orthodontics, Indira Gandhi Institute of Dental Sciences, Sri Balaji Vidyapeeth (Deemed to be University), Pondicherry, IND; 2 Orthodontics, Dr. M.G.R. Educational and Research Institute, Chennai, IND; 3 Conservative Dentistry and Endodontics, Dr. M.G.R. Educational and Research Institute, Chennai, IND; 4 Orthodontics, Sri Venkateswara Dental College, Chennai, IND; 5 Orthodontics, RVS Dental College and Hospital, Coimbatore, IND

**Keywords:** lingual orthodontics, periodontitis, plaque

## Abstract

Aim

To compare the periodontal status in relation to the lower anteriors of patients between labial and lingual orthodontic therapy.

Materials and methods

The study includes a total of 20 patients in the age group of 20-30 years. All the included patients were selected with limited lower anterior crowding within 0-8 mm. The subjects were randomly divided into two groups: labial (n=10) and lingual (n=10) fixed orthodontic therapy. The periodontal status was evaluated using three indices, plaque index, calculus index, and gingival index, at two different treatment intervals - the first month and the third month - of orthodontic treatment.

Results

The values of all the three indices at both time intervals were tabulated. There was no statistical significance when compared to the values in the first month. In the third month, all three indices were statistically significant for both labial and lingual therapy (p<0.001). The lingual appliance had more plaque and calculus accumulation.

Conclusion

Therefore, the study proves that the lingual surface of patients undergoing lingual orthodontic treatment exhibits more plaque and calculus deposition, thereby the weakening of the periodontal status.

## Introduction

Orthodontic treatment should be beneficial for the patient by improving his/her oral health, occlusion, aesthetics, and appearance [1]. The labial approach fixed orthodontic appliance facilitates the delivery of the desired force, allowing a more convenient approach for the manipulation of the appliance. The need for fixing the attachments on the lingual surfaces, better known as lingual orthodontics, has evolved in an attempt to address the aesthetic concerns, especially among the adult patients, due to psychosocial reasons. Lingual orthodontics came into existence as a viable option for orthodontic patients in the 1970s.

The maintenance of oral hygiene is quite a challenge in fixed orthodontic therapy [2-3]. There is a qualitative shift of the microbial flora that affects the integrity of hard and soft tissues [4-6]. The material and surface properties of the bracket can influence bacterial attachment, plaque-retaining capacity, and microbial diversity. Little is known about the adherent subgingival environment around orthodontic bands and the potential of mucosal components to prevent long-term subgingival biofilm formation on these surfaces. Therefore, the purpose of the present research was to perform a quantitative analysis of periodontal status for a three-month follow-up period in patients undergoing labial and lingual orthodontic therapy using the periodontal index (plaque index, calculus index, and gingival index).

## Materials and methods

This study was designed as a prospective clinical trial. The study was conducted in the Department of Orthodontics, Thai Moogambigai Dental College, Dr MGR Educational and Research Institute, after obtaining institutional ethical committee clearance (Dr.M.G.R DU/TMDCH/2018-19/18219002). Informed consent was obtained from all the patients recruited for this study. The inclusion criteria were patients with a class I/II division I/II division II/class III malocclusion, with the crowding of the lower anterior teeth less than 8 mm, between the age group of 20 and 30-year-old. A total of 20 patients were selected based on the inclusion criteria. All the patients underwent complete ultrasonic scaling before orthodontic treatment. The exclusion criteria included patients with skeletal class II/III malocclusions, gross dentofacial abnormalities, a systemic illness or debilitating diseases, for whom lingual orthodontic treatment was contraindicated, and with poor oral hygiene or periodontal diseases.

The patients were segregated so as to allot an equal number of patients in both groups. Group I formed 10 patients for whom labial fixed orthodontic appliances with MBT 022” prescription (3M Victory series brackets; 3M, Saint Paul, Minnesota) were bonded. Group II formed 10 patients for whom lingual fixed orthodontic appliances were bonded (2D lingual brackets with 18”22” slot size JJ orthodontic brackets; JJ Orthodontics, Thrissur, Kerala). Three indices that would evaluate the gingival and periodontal health of lower anterior teeth were chosen (the plaque, calculus, and gingival indices). Since any values recorded immediately after bonding would not show significant scores, as oral prophylaxis was performed for every patient just prior to bonding, the indices were not recorded in the bonding appointment. The baseline values were recorded after one month of bonding (T0). The assessment of plaque, calculus, and gingival indices was done by an independent assessor (periodontist) who was not part of this study. The plaque, calculus, and gingival index scores were recorded based on the previously proposed criteria [7-9]. After bonding, the patients were instructed to use the standard oral hygiene kit, which included an orthodontic manual toothbrush, chlorhexidine mouthwash, and interdental brush.

The second values of the plaque, calculus, and gingival indices were recorded at the end of the third month by the same assessor (T1). No prophylactic procedures were performed during this time interval between T0 and T1 so as to reduce any discrepancy in index scores. Oral hygiene instructions were reinforced at treatment visits between T0 and T1 for patients in both groups.

Statistical analysis

The values thus recorded were tabulated and assessed for descriptive and inferential statistics. The results of normality tests, Kolmogorov-Smirnov and Shapiro Wilks, showed that the variables did not follow a normal distribution. Therefore, to analyze the data, non-parametric tests were applied. The Mann-Whitney test was used to compare the indices scores between Group I and Group II patients and the p-value was set at <0.005.

## Results

In the present prospective clinical trial, plaque, calculus, and gingival scores were recorded in both groups, for example, labial and lingual orthodontic therapy at the end of the first and third months. The Mann-Whitney test was used to compare the value of all the three indices. All three indices were not statistically significant at the end of the first month (Table 1). However, the plaque, calculus, and gingival indices scores were significant at the third month (p<0.001) (Table 2). The lingual appliance had more plaque and calculus accumulation.

**Table 1:** Comparing all the three indices for labial and lingual therapy at the end of the first month

Index	Group	Mean	S.D.	Mean rank	P-value
Plaque index	Labial	1.0	0.8	64.10	0.223
	Lingual	1.0	0.7	56.90	
Calculus index	Labial	0.9	0.8	62.30	0.545
	Lingual	0.7	0.6	58.70	
Gingival index	Labial	1.3	0.8	68.60	0.006
	Lingual	0.8	0.6	52.40	

**Table 2:** Comparing all the three index for both labial and lingual therapy at the third month

Index	Group	Mean	S.D.	Mean rank	P-value
Plaque index	Labial	1.1	0.9	43.10	<0.001
	Lingual	1.8	0.9	77.90	
Calculus index	Labial	1.4	0.8	41.00	<0.001
	Lingual	1.6	0.9	80.00	
Gingival index	Labial	1.7	0.5	42.50	<0.001
	Lingual	1.8	0.8	78.50	

## Discussion

The ultimate goal of orthodontic therapy is to improve facial esthetics and function. Indirectly, it also addresses the health of supporting structures [10]. A recent systematic review analyzed 13 studies; it concluded that the levels of subgingival periodontal pathogens increased after orthodontic appliance placement and appeared to return to the pretreatment levels several months later [11]. Placement of orthodontic brackets influences the accumulation and composition of the supragingival and subgingival microbiota [12]. The success of orthodontic treatment, both short and long term, are strongly influenced by the patient’s periodontal status and post-treatment maintenance by the patient. The periodontal pathobiology is a multifactorial process and the orthodontist must clearly recognize all the clinical presentations of inflammatory periodontal diseases. Co-operation between various specialties in dentistry is of utmost importance in establishing correct diagnosis and treatment planning. One such inter-department interaction always exists between dentofacial-orthodontics and periodontics. There are many cases presented in the literature, which clearly shows that periodontal health is improved by orthodontic tooth movement, whereas orthodontic tooth movement can also be often facilitated by periodontal treatment.

‘‘Invisible’’ orthodontic treatment can be provided to the patients by using fixed lingual orthodontic therapy. Due to the increasing esthetic demands of adolescent patients, the indication of lingual orthodontic therapy is growing [13]. In the past, a prefabricated lingual bracket appliance was reported to cause problems in clinical application due to difficult bracket and archwire insertion. Furthermore, subjective impairments were reported by the patients such as oral discomfort as a result of injury to the tongue, speech dysfunction as a result of a restricted functional space for the tongue, and restriction of mastication. Since there is a change in oral bacteria after orthodontic bracket placement, this clinical trial was planned. Although there are a few studies comparing periodontal status after lingual and labial orthodontic therapy, there is still a lack of clear evidence. The majority of patients enrolled in the present study belonged to the age group of 20-30 years. Stadelmann et al. found that almost a quarter of the population undergoes orthodontic treatment, half of it with a mean age of 15 to 24 years old [14].

Now, comparing the two groups of patients in our study, we can see that the lingual group demonstrates more difficulty in removing food and plaque deposits around the brackets, as confirmed by the literature [15-16]. In the labial group from the scores of all three indices, one could clearly infer that there were initial gingivitis and plaque formation in the first month. In the third month, there was a slight worsening of the oral hygiene status in the labial group. However, there was no statistical significance among the two time periods in the labial group. When lingual group scores were analyzed in comparison to the labial group; the lingual group had more oral hygiene impairment. According to many retrospective and prospective studies of the literature, wider lingual brackets cause a reduced inter-bracket distance and make oral hygiene procedures very difficult, with consequent risk for plaque accumulation and gingivitis [16-17].

Oral hygiene instruction would be essential in all cases of orthodontic treatment and the use of adjuncts such as sonic electric toothbrushes, interproximal brushes, chlorhexidine mouthwashes, fluoride mouthwashes, and regular professional cleaning should be reinforced. However, patient motivation and dexterity are paramount in the success. Saliva is an important modifying factor in the cariogenic potential of dental plaque; thus, it should be considered in orthodontic treatment planning. Hence, in future research, it is necessary to evaluate the salivary flow, buffer capacity, and Streptococcus mutans count for a longer follow-up period.

## Conclusions

Within the limitations of this prospective clinical trial, it could be concluded that both the lingual and labial surfaces showed plaque and calculus accumulation and a change in periodontal conditions. The lingual surface of the patients undergoing lingual orthodontic treatment exhibits more plaque and calculus deposition and the weakening of periodontal status. The reason attributed for the increase in plaque formation in the lingual group was said to be the design of the appliance. Hence, future directions of research could be directed towards improving the design of the lingual appliance. Since both groups had plaque accumulation during the follow-up period, it is of utmost importance to include oral hygiene instruction as part of the treatment protocol. This could clearly improve the outcome of orthodontic treatment. Future clinical trials should be planned with an extended follow-up period.

## References

[1] Lombardo L, Ortan YO, Gorgun O, Panza C, Scuzzo G, Siciliani G (2013). Changes in the oral environment after placement of lingual and labial orthodontic appliances. Prog Orthod.

[2] Zachrisson S, Zachrisson BU (1972). Gingival condition associated with orthodontic treatment. Angle Orthod.

[3] Zachrisson BU (1972). Gingival condition associated with orthodontic treatment. II. Histologic findings. Angle Orthod.

[4] Diamanti-Kipioti A, Gusberti FA, Lang NP (1987). Clinical and microbiological effects of fixed orthodontic appliances. J Clin Periodontol.

[5] Naranjo AA, Trivino ML, Jaramillo A, Betancourth M, Botero JE (2006). Changes in the subgingival microbiota and periodontal parameters before and 3 months after bracket placement. Am J Orthod Dentofacial Orthop.

[6] Kim K, Heimisdottir K, Gebauer U, Persson GR (2010). Clinical and microbiological findings at sites treated with orthodontic fixed appliances in adolescents. Am J Orthod Dentofacial Orthop.

[7] Silness J, Loe H (1964). Periodontal disease in pregnancy II. Correlation between oral hygiene and periodontal condition. Acta Odontol Scand.

[8] Hinrichs JE (2003). The role of dental calculus and other predisposing factors. Clinical Periodontology. 9th ed.

[9] Loe H, Silness J (1963). Periodontal disease in pregnancy I. Prevalence and severity. Acta Odontol Scand.

[10] Shekar S, Bhagyalakshmi A, Chandrashekar BR, Avinash BS (2017). Periodontal considerations during orthodontic treatment. Indian J Oral Health Res.

[11] Guo R, Lin Y, Zheng Y, Li W (2017). The microbial changes in subgingival plaques of orthodontic patients: a systematic review and meta-analysis of clinical trials. BMC Oral Health.

[12] Franch M, De La Iglesia F, Auladell A, Walter A, Clusellas N, Puigdollers A (2019). Comparison of lingual and buccal orthodontic therapy on microbial parameters and periodontal status. J Med Biomed App Sci.

[13] Creekmore T (1989). Lingual orthodontics—its renaissance. Am J Orthod Dentofacial Orthop.

[14] Stadelmann P, Zemp E, Weiss C, Weiger R, Menghini G, Zitzmann NU (2012). Dental visits, oral hygiene behaviour, and orthodontic treatment in Switzerland. Schweiz Monatsschr Zahnmed.

[15] Caniklioglu C, Ozturk Y (2005). Patient discomfort: a comparison between lingual and labial fixed appliances. Angle Orthod.

[16] Stamm T, Hohoff A, Ehmer U (2005). A subjective comparison of two lingual bracket systems. Eur J Orthod.

[17] Miyawaki S, Yasuhara M, Koh Y (1999). Discomfort caused by bonded lingual orthodontic appliances in adult patients as examined by retrospective questionnaire. Am J Orthod Dentofacial Orthop.

